# What's Your Diagnosis? Dog With a Laryngeal Mass

**DOI:** 10.1111/vcp.70014

**Published:** 2025-06-04

**Authors:** Benjamin J. Haythornthwaite, Dylan Yaffy, Emma J. Holmes

**Affiliations:** ^1^ Royal Veterinary College Hatfield UK

**Keywords:** canine, immunohistochemistry, larynx, neoplasia

## 1 Case Presentation

**FIGURE 1:**
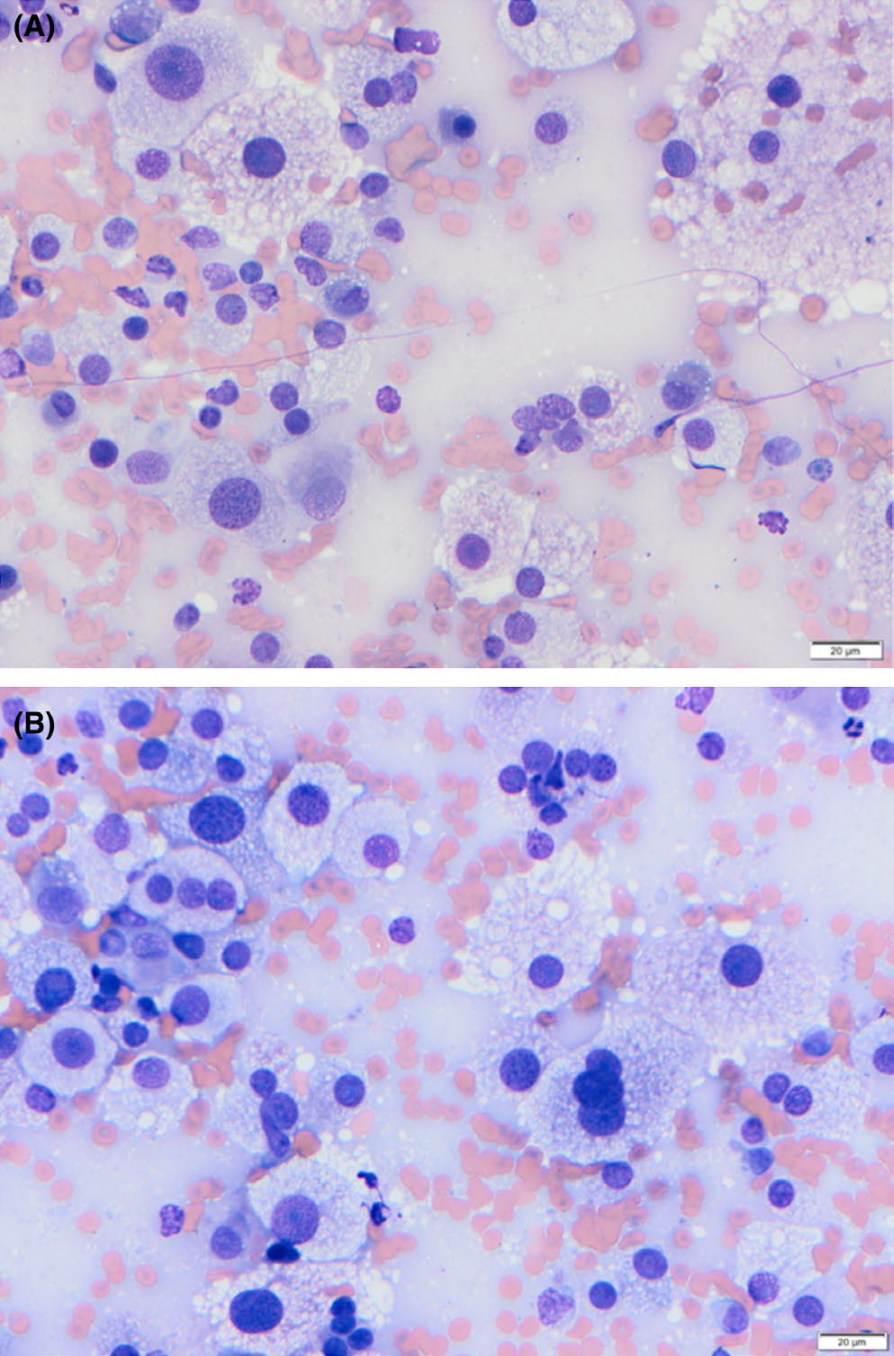
(A) Cytologic preparation of a laryngeal mass aspirate. Modified Wright stain, ×40. (B) Cytologic preparation of a laryngeal mass aspirate. Modified Wright stain, ×40.

**FIGURE 2:**
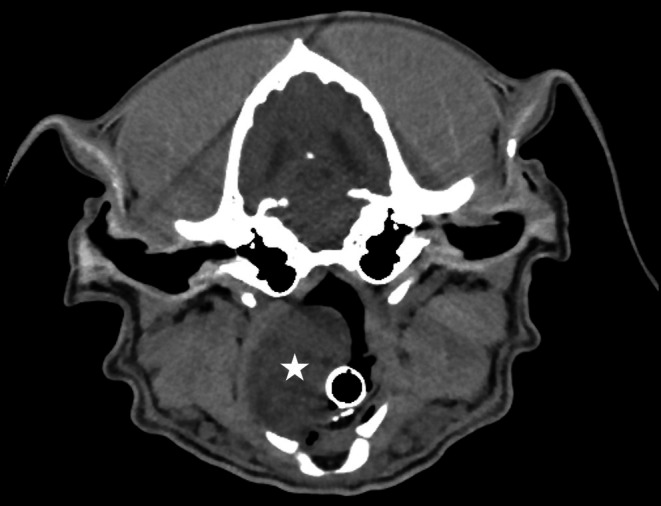
Transverse magnetic resonance imaging image of a mass (star symbol) displacing the laryngeal lumen of a dog.

An 8‐year, 3‐month‐old female, entire Boxer dog presented to the Queen Mother Hospital for Animals (QMHA), Royal Veterinary College (RVC) for evaluation of increased upper airway noise and dysphagia. A laryngeal mass had been identified by the referring veterinarian 5 months previously while the dog was undergoing general anesthetic for removal of oral masses (confirmed as peripheral odontogenic fibromas). At this time, no clinical signs were associated with the mass. However, in the weeks prior to presentation, the dog became unable to tolerate solid food and started showing respiratory symptoms relating to the mass and was subsequently referred for evaluation. CT examination revealed a 35 × 28 × 39 mm soft tissue mass occupying the right side of the rima glottidis, displacing the laryngeal lumen. The mass extended from the level of the rostral margin of the tympanic bulla to the level of C1 and dorsally into the caudal aspect of the nasopharynx. Direct aspirate preparations (Figures 1 and 2) from the laryngeal mass were air‐dried, fixed, and stained using modified Wright stain (Hema‐tek 3000, Siemens).

### 1.1 Cytologic Interpretation

#### 1.1.1 Malignant Neoplasia; Origin Not Fully Determined

Preparations were highly cellular, with low to moderate numbers of erythrocytes and low to moderate numbers of lysed cells on a pale blue background. The predominant nucleated population was variably sized round cells with round nuclei (approximately 1–2 RBCs in diameter), a coarsely stippled to clumped chromatin pattern, a prominent nucleolus, and variable amounts of highly granular to vacuolated, clear to pale pink cytoplasm, frequently with a fine dusting of pink granules. There was marked anisocytosis, moderate to marked anisokaryosis, mild intracellular anisokaryosis, frequent bi‐, tri‐, and multinucleation (up to 15–25 nuclei), multiple nucleoli, frequent macronuclei and macronucleoli, occasional nuclear molding, and rare mitotic figures. Low numbers of smaller round cells with round to oval nuclei, a coarsely stippled chromatin pattern, variably distinct nucleoli, and moderate amounts of mid‐pink cytoplasm, which was much less granular than that of the previously described population, were also scattered throughout (Figure 1).

Based on the location and morphology of the smaller, less pleomorphic cells, differential diagnoses including oncocytoma, rhabdomyoma, or granular cell tumor were considered. Given the frequent malignant features, other differential diagnoses, such as histiocytic sarcoma, rhabdomyosarcoma, anaplastic carcinoma, amelanotic melanoma, and salivary carcinoma, were also suggested.

## 2 Additional Results

Incisional biopsy samples obtained at the same time as the aspirates were fixed in formalin, routinely processed, and stained with H&E.

**FIGURE 3:**
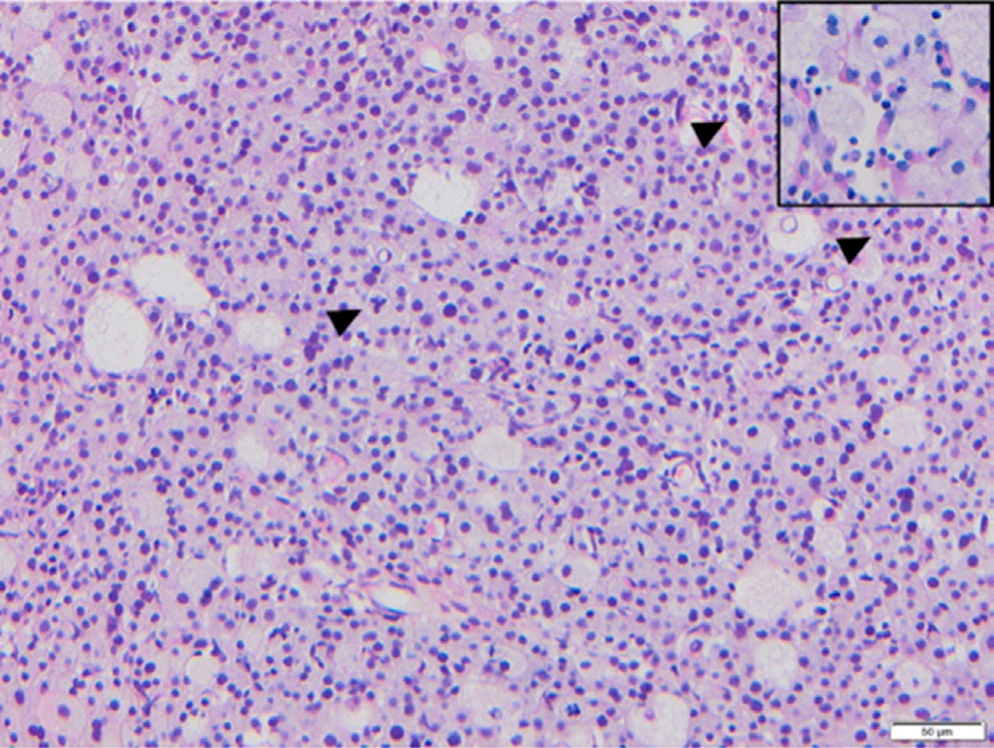
Photomicrographs of histologic sections from a laryngeal rhabdomyosarcoma in a dog, H&E, ×20 objective. Diffuse sheet of atypical malignant neoplastic cells with a large amount of vacuolated wispy cytoplasm and numerous bizarre mitoses (arrowheads). Inset: PAS, ×40 objective. Smaller, less vacuolated neoplastic cells contain intracytoplasmic PAS‐positive material.

Sections had a densely cellular pleomorphic malignant neoplasm, composed of diffuse sheets of irregularly polygonal to elongate cells of two populations. The more prominent population had large amounts of vacuolated cytoplasm with lower numbers of smaller round cells with moderate amounts of finely granular eosinophilic cytoplasm. Most cells had a single irregularly round nucleus with one prominent large nucleolus. Anisocytosis and anisokaryosis of the larger cells were marked, with frequent multinucleated giant cells and 57 bizarre mitotic figures in 2.37 mm^2^ (Figure 3). Atypia of the smaller cell population was mild. The histological interpretation was an atypical malignant neoplasm with differentials including rhabdomyoma/sarcoma, oncocytoma, granular cell tumor, amelanotic melanoma, or histiocytic sarcoma.

**FIGURE 4:**
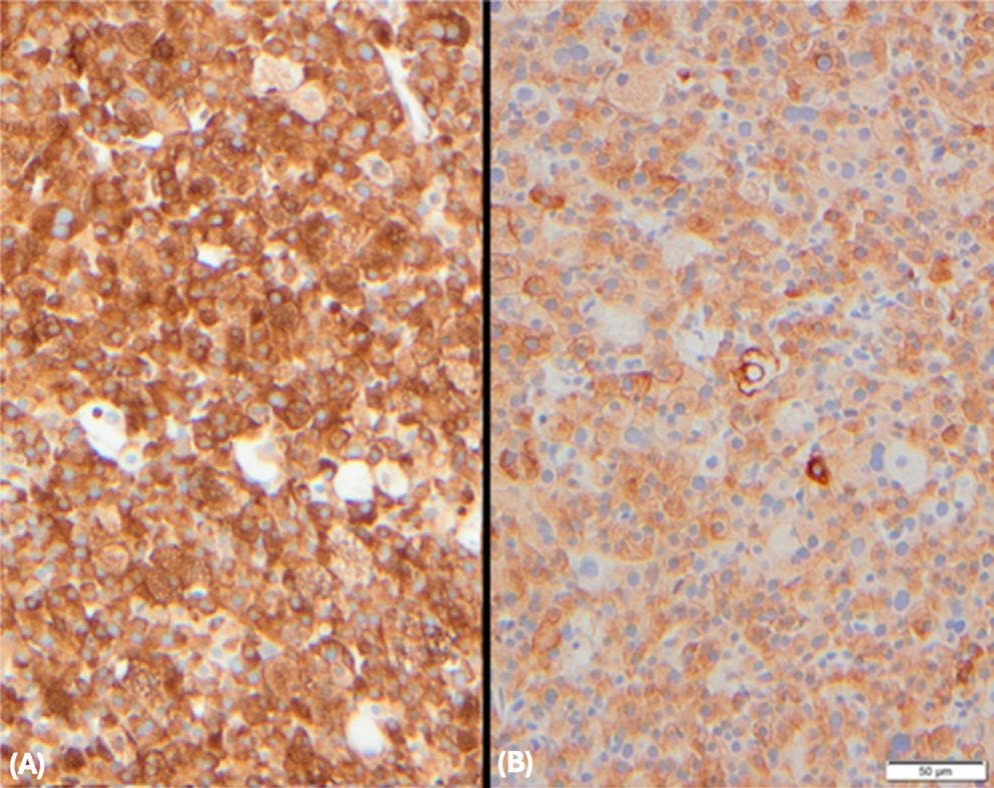
Photomicrographs of histologic sections from a laryngeal rhabdomyosarcoma in a dog, immunohistochemistry, vimentin (A) and desmin (B), ×20 objective. Neoplastic cells, including multinucleated and atypical cells, exhibit diffuse strong membranous and cytoplasmic labeling with antibodies against vimentin and moderate to focally strong cytoplasmic labeling against desmin.

Histochemical staining revealed strong cytoplasmic positivity for periodic acid‐Schiff (PAS) in the small cells but negativity in the larger cells, diffuse strong membranous, and cytoplasmic positivity for vimentin in approximately 95% of cells (Figure 4), diffuse moderate cytoplasmic positivity for desmin in approximately 80% of cells, and negativity for toluidine blue, phosphotungstic acid hematoxylin (PTAH), cytokeratin, ionized calcium‐binding adaptor molecule 1 (Iba‐1), melan‐A, smooth muscle actin (α‐sma), neuron‐specific enolase (NSE), and S100 (Table 1).

**TABLE 1:** Immunohistochemical staining characteristics of neoplastic cells from a laryngeal mass.

Additional staining	Positive	Staining location	Negative
PAS	Small cells	Cytoplasmic	Large cells
Toludine blue			X
PTAH			X
Cytokeratin			X
Vimentin	Strong	Cytoplasmic and membranous	
Iba‐1			X
Melan‐A			X
Desmin	Strong	Cytoplasmic	
SMA			X
NSE			X
S100			X

Abbreviations: α‐sma, smooth muscle actin; Iba‐1, ionized calcium‐binding adaptor molecule 1; NSE, neuron‐specific enolase; PAS, periodic acid‐Schiff; PTAH, phosphotungstic acid hematoxylin.

Based on these staining characteristics and cellular pleomorphism, a diagnosis of rhabdomyosarcoma was made.

## 3 Discussion

Laryngeal rhabdomyosarcoma is mainly reported in adult dogs, being locally invasive but rarely metastatic. This local invasion makes complete excision difficult, with tumor recurrence often resulting in euthanasia [1, 2].

Without immunohistochemical (IHC) staining characteristics, diagnosis can be difficult due to cellular pleomorphism or the presence of multiple distinct cellular populations as demonstrated in this case [3, 4]. The range of cellular morphologies is suspected to resemble various developmental phenotypes of the myocyte, from round immature myoblasts with variable amounts of pink cytoplasm to more mature multinucleated myotubular cells [1]. The description of the immature myoblasts appears to correlate with the small pink cells described in the current case; however, the large, highly granular to vacuolated cells often exhibiting marked cellular pleomorphism made the cytological diagnosis highly challenging.

Rhabdomyosarcomas can be differentiated histologically into four subclasses, including alveolar, botryoid, embryonal, and rarely pleomorphic rhabdomyosarcoma, allowing prediction of biological behavior. Alveolar RMS are further classified into classic and solid types, with embryonal separated into rhabdomyoblastic (dominated by round to polygonal cells with variable pink cytoplasm), myotubular (predominance of multinucleated PTAH positive “strap‐cells”), and spindled which is rare in veterinary species [1, 3].

IHC characteristics are often variable, depending on the degree of cellular differentiation and the variability of myogenic regulating factor expression. Vimentin is the first intermediate filament expressed at the myoblast stage, with further differentiation resulting in desmin expression. Desmin can be useful, even in undifferentiated and highly pleomorphic RMS. Expression of this marker with concurrent lack of cytokeratin and PAS expression would exclude a diagnosis of oncocytoma [5]. Ideally, for further confirmation of skeletal muscle origin, MyoD1 and myogenin expression, representing early embryonal transcription factors, could have been considered, although expression may be variable depending on the degree of differentiation [1]. Unfortunately, these markers were not available at our institution. Negative smooth muscle actin (SMA) staining excluded smooth muscle origin. Despite the highly granular morphology and PAS positivity, a granular cell tumor was excluded based on S100 and NSE negativity [5]. Findings were also not compatible with histiocytic sarcoma (Iba‐1 negativity), amelanotic melanoma (melan‐A and S100 negativity), or anaplastic carcinoma (cytokeratin negativity).

The current case ultimately demonstrates an uncommon neoplasm, which provided a diagnostic challenge given the degree of cellular pleomorphism, ultimately requiring immunohistochemistry for diagnosis.

## Conflicts of Interest

The authors declare no conflicts of interest.

## References

[1] B. G. Caserto , “A Comparative Review of Canine and Human Rhabdomyosarcoma With Emphasis on Classification and Pathogenesis,” Veterinary Pathology 50, no. 5 (2013): 806–826. 23439712 10.1177/0300985813476069

[2] A. Gombert , D. Culand , I. Lanthier , et al., “Two Concurrent Embryonal Rhabdomyosarcomas of the Oesophageal and Perilaryngeal Tissue in a Dog: Imaging, Cytological and Histological Features,” Veterinary Record Case Reports 8, no. 3 (2020): e001119.

[3] M. Murakami , H. Sakai , N. Iwatani , et al., “Cytologic, Histologic and Immunohistochemical Features of Maxillofacial Alveolar Rhabdomyosarcoma in a Juvenile Dog,” Veterinary Clinical Pathology 39 (2010): 113–118. 19645742 10.1111/j.1939-165X.2009.00175.x

[4] K. Barnhart and B. Lewis , “Laryngopharyngeal Mass in a Dog With Upper Airway Obstruction,” Veterinary Clinical Pathology 29 (2000): 47–50. 12070810 10.1111/j.1939-165x.2000.tb00397.x

[5] T. S. Rallis , D. K. Tontis , N. H. Soubasis , K. K. Patsiaura , L. G. Papazoglou , and K. K. Adamama‐Moraitou , “Immunohistochemical Study of a Granular Cell Tumor on the Tongue of a Dog,” Veterinary Clinical Pathology 30 (2001): 62–66. 12024318 10.1111/j.1939-165x.2001.tb00260.x

